# Cooperative activation of carbon–hydrogen bonds by heterobimetallic systems

**DOI:** 10.1039/d3dt03571a

**Published:** 2023-12-21

**Authors:** Abdelhak Lachguar, Andrey V. Pichugov, Till Neumann, Zachary Dubrawski, Clément Camp

**Affiliations:** a Université de Lyon, Institut de Chimie de Lyon, Laboratory of Catalysis, Polymerization, Processes & Materials, CP2M UMR 5128 CNRS-UCB Lyon 1-CPE Lyon 43 Bd du 11 Novembre 1918 F-69616 Villeurbanne France clement.camp@univ-lyon1.fr

## Abstract

The direct activation of C–H bonds has been a rich and active field of organometallic chemistry for many years. Recently, incredible progress has been made and important mechanistic insights have accelerated research. In particular, the use of heterobimetallic complexes to heterolytically activate C–H bonds across the two metal centers has seen a recent surge in interest. This perspective article aims to orient the reader in this fast moving field, highlight recent progress, give design considerations for further research and provide an optimistic outlook on the future of catalytic C–H functionalization with heterobimetallic complexes.

## Abstract 2

The activation of C–H bonds by heterobimetallic compounds is a rich area of research that has recently received increased attention. This perspective highlights recent advances and aims to guide the reader in this rapidly evolving field.

## Graphical abstract

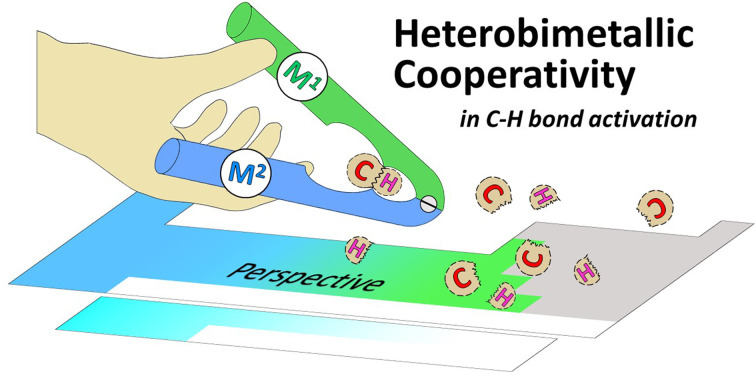

## 1. Introduction

**Fig. 1:**
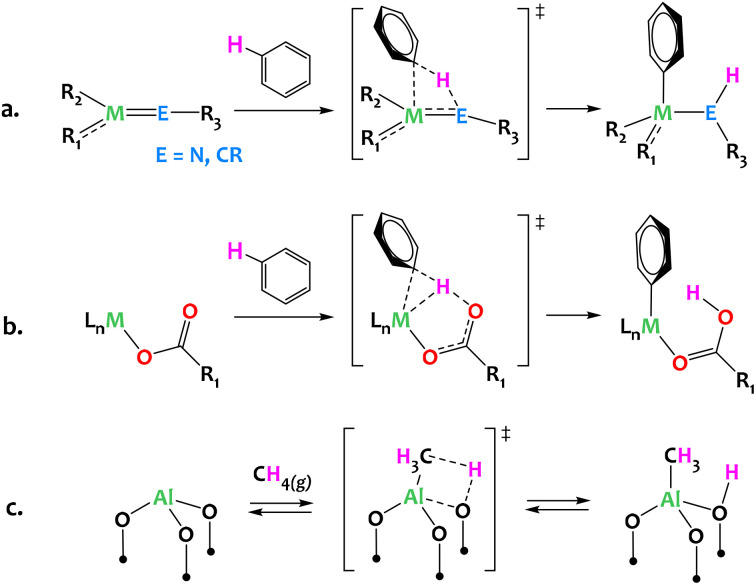
Representative activation of carbon hydrogen bonds *via* heterolytic mechanisms: (a) 1,2-addition across early metal imides or alkylidynes;^1–11^ (b) Concerted Metalation–Deprotonation (CMD) mechanism typical of late metal systems associated with bases (carbonates, acetates, hydroxyls *etc*.);^12–18^ (c) dissociative adsorption of methane across the Al–O moieties of dehydrated γ-alumina.^19,20^

Often considered the “holy grail” of organometallic chemistry, direct C–H activation is a key organic transformation with well-proven industrial applicability. Given the Green Chemistry principles, direct C–H activation can provide a critical path forward through atom economic synthetic strategies and minimization of hazardous wastes. Established mechanisms in organometallic chemistry, such as oxidative addition or σ-bond metathesis, have historically been critical to any form of C–H functionalization. However, multi-partnered activation *via* heterolytic pathways provides exciting new opportunities for efficient C–H activation with well-established mechanisms involving metal and non-metal atoms (Fig. 1). Classic examples which fall into this category include 1,2-addition/elimination to metal-element multiple bonds, such as early metal imides and alkylidenes/alkylidynes (Fig. 1a),^1–11^ and concerted metalation deprotonation (CMD), wherein late metal alkane σ-complexes weaken the C–H bond such that deprotonation can occur from nearby weak bases (Fig. 1b).^12–18^ In the context of heterogeneous catalysis, (Al^III^, O) Lewis acid–base pairs of γ-alumina are proposed to activate methane in a heterolytic C–H bond cleavage across surface Al^III^–O motifs (Fig. 1c).^19,20^ Similarly, several propane dehydrogenation heterogeneous catalysts are thought to operate through a rate-determining heterolytic C–H activation step *via* a 1,2 addition across M–O surface sites (M = Cr, Co).^21,22^

Metal complexes paired with main group elements are clearly an effective strategy however, exchanging the main group center for a second metal can give significant advantages; coordination number, geometry and redox properties can all be tuned with judicious choice of a second metal center and surrounding ligands. The presence of d-orbitals within this secondary metal also allows for textbook organometallic steps to occur with both partners and thus bimetallic complexes could offer ample opportunities for complex C–H functionalization catalysis.^23,24^

Accordingly, there has been a distinct surge of interest in the preparation and reactivity of heterobimetallic complexes in the last decade, driven by the hope that two metal centers are better than one. Several successes have been realized with bimetallic species activating several small molecules such as O_2_,^25–28^ H_2_ ^29–34^ and CO_2_.^35–41^ However, the development of systems able to activate and transform C–H bonds in a concerted way, across two metal centers, is still limited. Therefore, this perspective intends to shine light on recent progress in heterobimetallic C_sp/sp^2^/sp^3^_–H bond activation and discusses important mechanistic insights toward competent catalytic processes.

This perspective is not meant to be exhaustive, but will discuss key examples to illustrate the diversity of chemistries offered by this rapidly evolving area of research, with the hope of stimulating progress and inspiring new chemistry. Homobimetallic systems, even when two metal centers are at play, are not considered. Interested readers are directed to the many excellent articles previously written on these subjects.^29,42–45^

## 2. Early/late transition metal systems

Perhaps the most widely explored series are the early/late heterobimetallic systems. The combination of two transition metal atoms from opposite sides of the d-block in a single molecule can often result in highly polarized metal–metal interactions.^46,47^ As a consequence of this polarization, the interaction of early/late systems with substrates can also be highly asymmetrical, which can be used to trigger the heterolytic cleavage of C–H bonds.

**Fig. 2:**
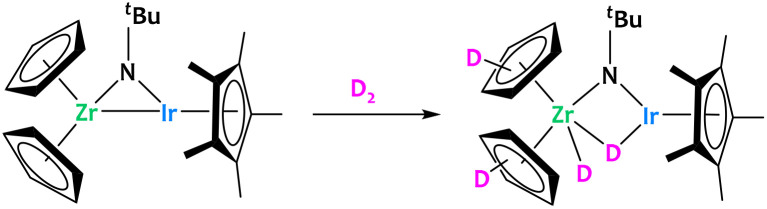
Reactivity of a Zr/Ir complex, showing deuterium incorporation on the Cp rings.^48^

The pioneer example of such reactivity was reported by Bergman using the bridging imido Zr/Ir complex, [Cp_2_Zr(μ-N^*t*^Bu)IrCp*]. This complex, bearing two coordinatively unsaturated metal centers bonded together, was shown to activate a series of substrates containing polar and non-polar bonds.^48–52^ Within the context of this review, it is interesting to note that after addition of D_2_ to this Zr/Ir complex, deuterium was found to be incorporated into the Cp rings (Fig. 2). This shows that this heterobimetallic system is competent for C–H activation.^48^

**Fig. 3:**
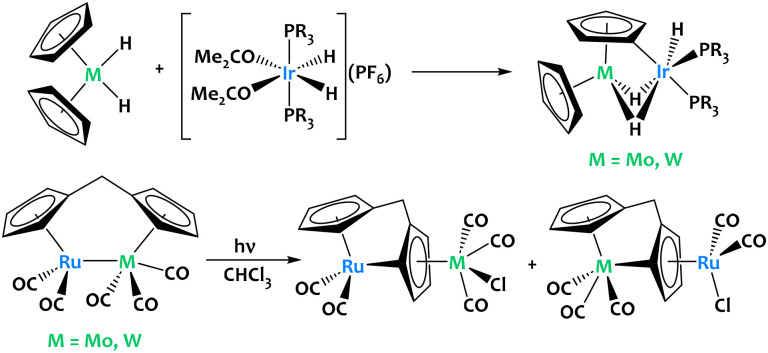
Stoichiometric activation of cyclopentadienyl C–H bonds in Ir–M and Ru–M complexes (M = Mo, W).^53–55^

Stoichiometric activation of cyclopentadienyl C–H bonds were also reported in ruthenium-group 6 and iridium-group 6 heterobimetallic species (Fig. 3). Although cooperative reactivity is not definitely proven in these early examples, they highlight the potential of early/late heterobimetallic complexes for C–H bond activation.

**Fig. 4:**
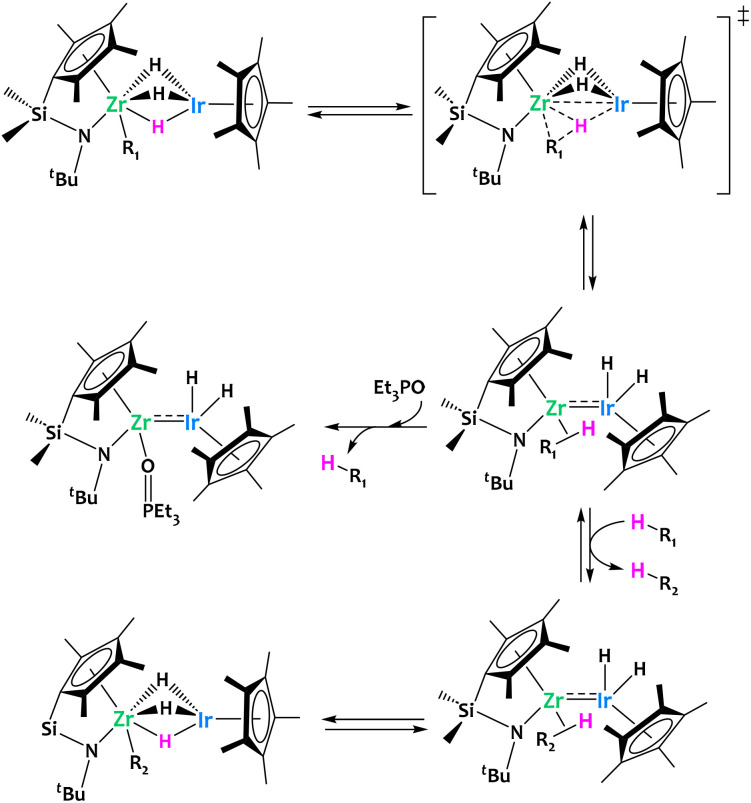
Cooperative C–H activation by a Zr/Ir complex.^56,57^

As a continuation of previous works on heterobimetallic complexes of group 4 metals and Ir,^57,58^ in 2014 Suzuki and coworkers reported the preparation of Zr/Ir polyhydrido complexes supported by ansa-(cyclopentadienyl)amide ligands (Fig. 4).^56^ These complexes were shown to promote the reversible C–H activation across the two metals of a rich scope of aliphatic and aromatic substrates at moderate temperatures. Detailed mechanistic investigations suggested that the intermediate formation of unsupported multiple bonds between Zr and Ir is of critical importance in this reactivity. This hypothesis is supported by DFT calculations and the trapping of a Et_3_PO–adduct featuring a Zr/Ir unsupported metal–metal bond (Fig. 4). In addition, the authors successfully carried out the catalytic H/D exchange between C_6_D_6_ and a range of arenes in the presence of the aforementioned heterobimetallic Zr/Ir phenyl derivative, further highlighting the interest of such system for splitting C–H bonds.^56^

**Fig. 5:**
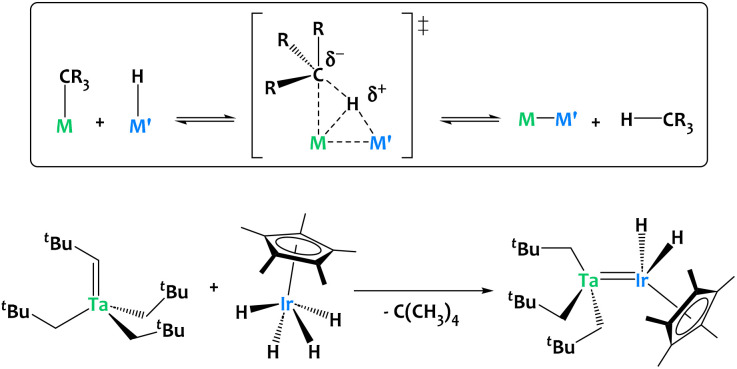
Alkane elimination synthesis of heterobimetallic species, exemplified on a Ta/Ir complex.^59–63^

Our group developed an alkane elimination synthetic strategy between nucleophilic metal alkyl derivatives and a Brønsted acidic iridium polyhydride to generate original heterobimetallic species (Fig. 5).^59–63^ Such systems are, by virtue of the principle of microscopic reversibility, particularly well poised to promote the reverse reaction, *i.e.* the heterolytic cleavage of C–H bonds through unusual cooperative metal–metal pathways (Fig. 5).

**Fig. 6:**
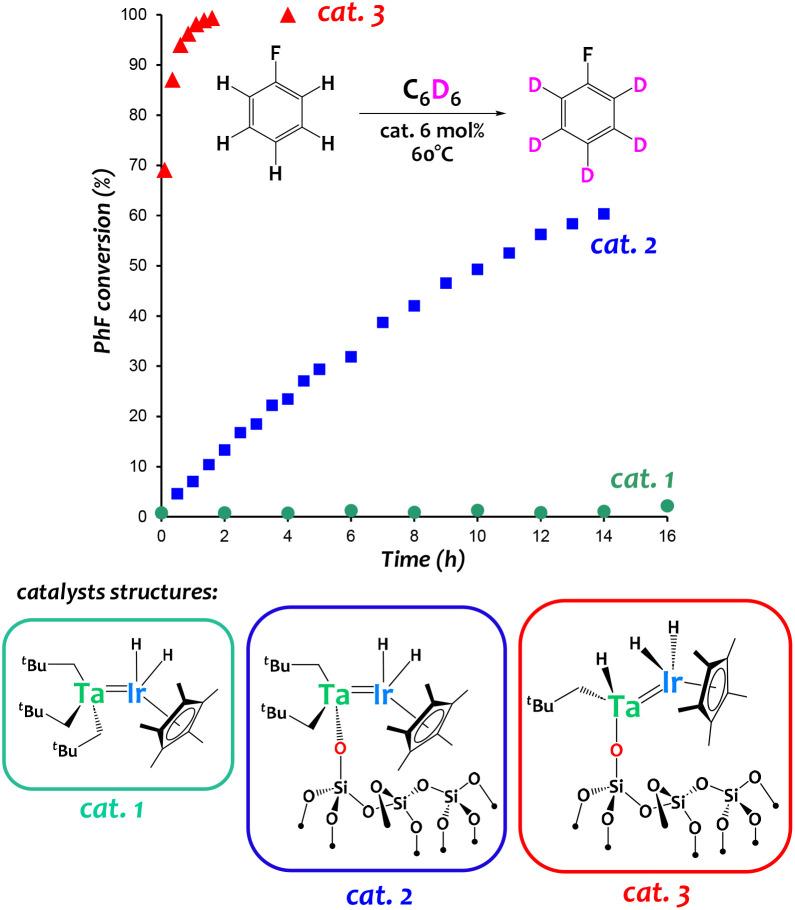
Catalytic performances of Ta/Ir species in an arene hydrogen isotope exchange reaction between C_6_D_6_ and fluorobenzene.^60,64^

Complex [{Ta(CH_2_^*t*^Bu)_3_}{IrH_2_(Cp*)}] (Fig. 6, catalyst 1) has been used to prepare well-defined silica-supported low-coordinate heterobimetallic polyhydrido species [≡SiOTa(CH_2_^*t*^Bu)_2_{IrH_2_(Cp*)}] (Fig. 6, catalyst 2) and [≡SiOTa(CH_2_^*t*^Bu)H{IrH_2_(Cp*)}] (Fig. 6, catalyst 3) using a Surface OrganoMetallic Chemistry (SOMC) approach.^60,61^ These original silica-supported species exhibit drastically enhanced catalytic performances in arene H/D exchange reactions with respect to (i) monometallic analogues (for instance, [(≡SiO)_2_Ta(H)_*n*_] was found much less active in this reaction)^60^ as well as (ii) homogeneous systems (cat. 1 in Fig. 6).^60^

**Fig. 7:**
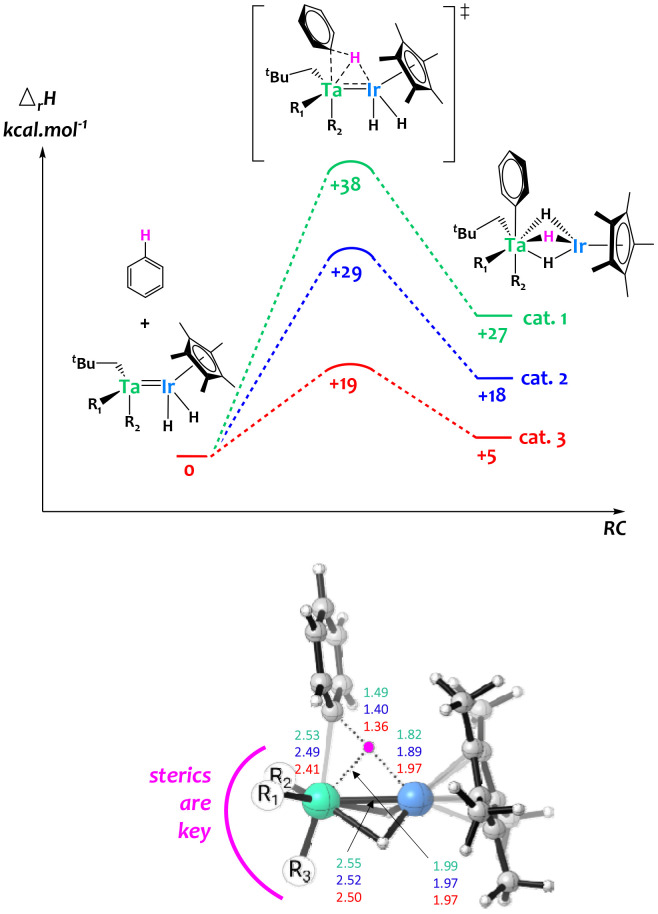
Top: computed enthalpy profile for the H–D exchange of benzene catalyzed by the Ta/Ir complexes 1, 2 and 3. Bottom: 3D representation of the computed transition state of the bimetallic C–H activation together with key distances (Å).^64^

Computational studies were carried out on these systems and suggest that steric constraints around the bimetallic cores play a critical role to synergistically activate C–H bonds across the two metals,^64^ explaining the remarkable H/D exchange catalytic activity observed in the less hindered surface organometallic Ta/Ir species. Notably, as shown in Fig. 7, the computed bimetallic C–H activation barrier follows the same order as the experimental activity, with catalyst 1 being the least active and catalyst 3 being the most active. The examination of the transition state geometry suggests that the heterobimetallic C–H bond cleavage is eased when the bimetallic core is sterically accessible, aiming to minimize the tantalum–carbon distance. This favors donation from the C–H σ orbital to an empty d orbital on Ta, while the iridium center is essential for providing nucleophilic assistance through back-donation into the C–H σ* orbital. This investigation highlights the advantages of the SOMC approach, enabling the creation of unique unsaturated species inaccessible in solution thanks to the solid support.

**Fig. 8:**
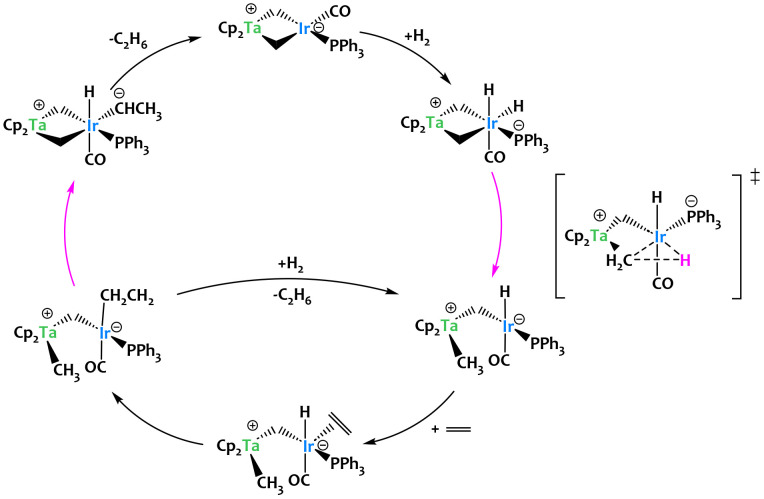
Alkyne hydrogenation catalyzed by a Ta/Ir complex. The key transition step to generate the active species is the cooperative C–H reductive elimination at the μ-CH_2_ group.^65–69^

Bergman and coworkers investigated catalytic alkene hydrogenation promoted by [E(CH_2_)_2_Ir(CO)(PPh_3_)] (E = Cp_2_Ta or Ph_2_P) complexes and discovered that the tantalum–iridium species can hydrogenate alkenes up to 150 times faster than the ylide analogue.^65–69^ Experimental and computational studies revealed that the heterobimetallic Ir–Ta interaction induces a catalytic mechanism (Fig. 8) that is different from the transition metal/main group Ir–P interaction. More specifically, the Ir/Ta complex is proposed to undergo much faster C–H reductive elimination of the hydride and μ-CH_2_ groups compared with the Ir/P analogue. The authors propose this is due to greater stabilization of the α-carbon anion character in the transition state by the cationic Cp_2_Ta moiety (Fig. 8). This key mechanistic step is the reverse of a concerted heterobimetallic C–H activation across the two metals, and does not occur in the Ir–P analogue.^65^ This study thus showcases the interest of early–late heterobimetallic compounds to promote alternative mechanistic pathways for the formation/cleavage of C–H bonds.

**Fig. 9:**
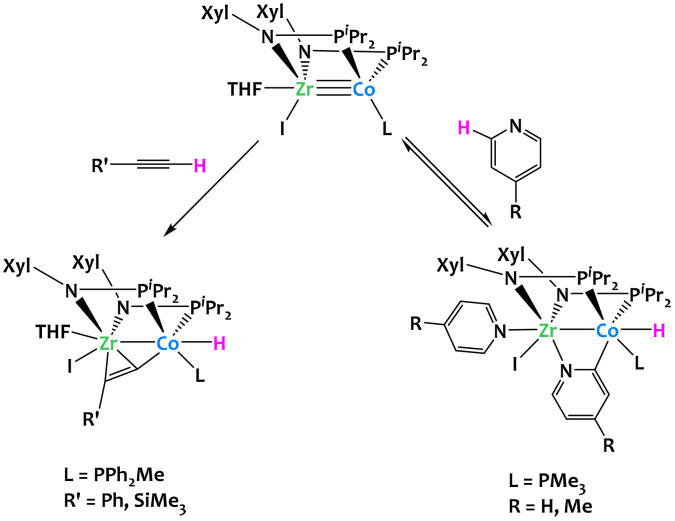
Cooperative C–H activation of pyridine derivatives and alkynes by a Zr/Co complex.^70^

C–H activation can also be achieved by heterobimetallic complexes based on first-row late metals. Thomas's research group demonstrated that bis(phosphinoamide) Zr^IV^/Co^−I^ heterobimetallic complexes can activate the *o*-C–H bonds of *para*-substituted pyridine derivatives and the terminal C–H bonds of terminal alkynes (Fig. 9).^70^ When the bis(phosphinoamide) Zr^IV^/Co^−I^ heterobimetallic [(THF)(I)Zr(XylNP^i^Pr_2_)_2_Co(L)] was treated with an excess of 4-methylpyridine, a displacement of the THF ligand was accompanied by an oxidative addition of the *o*-C–H bond of a second molecule of 4-methylpyridine, to generate a Zr^IV^/Co^I^ complex [(4-Me-C_5_H_4_N)(I)Zr(XylNP^i^Pr_2_)_2_(μ-4-Me-C_5_H_3_N)Co(L)(H)]. The same reactivity was observed with pyridine. The pyridine C–H activation process was found reversible, but this equilibrium could be displaced by the addition of an excess of pyridine. The bis(phosphinoamide) Zr^IV^/Co^−I^ heterobimetallic complex was also able to activate terminal alkyne C–H bonds. This resulted in the quantitative formation of the complex [(THF)(I)Zr(XylNP^i^Pr_2_)_2_(μ-PhCC)Co(PPh_2_Me)(H)], which contains a terminal cobalt hydride ligand and an alkyne η^2^-coordinated to the zirconium atom. In either case, the two metal centers react cooperatively to coordinate the substrate directing group (CC or N) to the Zr center and allow C–H activation to occur at the adjacent d^10^ Co^−I^ center.

**Fig. 10:**
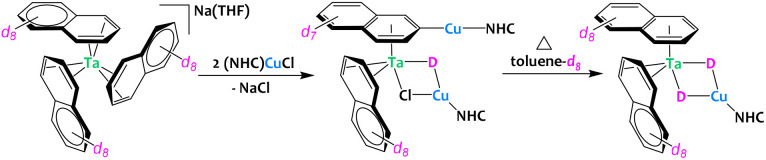
Heterometallic C–H activation of naphthalene *en route* to the formation of a Ta/Cu complex.^71^

Very recently, the group of Buss described the preparation of Ta/Cu architectures starting from a tris(naphthalene) tantalate complex (Fig. 10).^71^ During the synthesis, bridging hydrides are incorporated in these heterometallic compounds. The origin of these hydrides was investigated by isotopic crossover experiments using either deuterated solvent (THF-d_8_ or toluene-d_8_) and deuterated naphtalenide. The results suggest that one hydride originates from a naphthyl C–H bond activation event while the second hydride involves C–H activation of the solvent. Cooperative bimetallic C–H activation is proposed, yet the precise reaction mechanisms are not definitely proven and seem to be N-heterocyclic carbene dependent.

## 3. Late/late transition metal systems

Most focus has been placed onto the study of early/late heterobimetallic complexes due to their inherent asymmetry and polarity. However, late/late systems offer a unique advantage in that their wealth of d electrons can allow for π-backbonding into a substrate's π* orbitals, weakening the C–C or C–H bonds of the latter. Indeed, most examples in this section deal with unsaturated substrates such as alkenes and alkynes showing the interest of the electronic environment that late/late examples can offer.

**Fig. 11:**
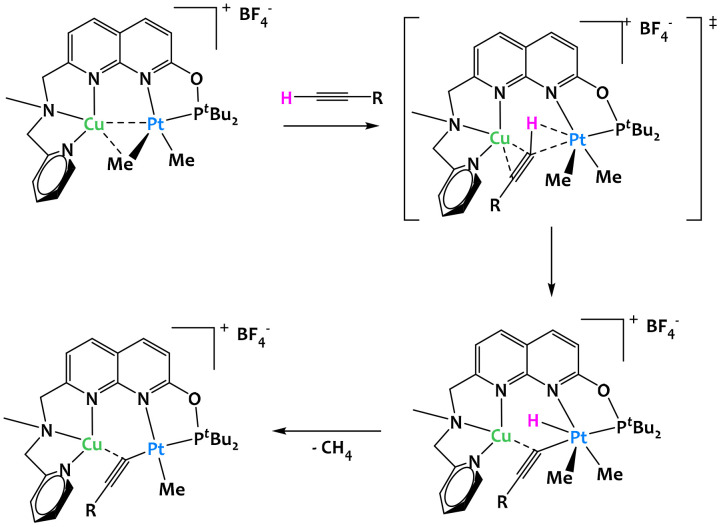
Cooperative C–H activation of terminal alkynes by a Cu/Pt complex.^72^

In 2020, Khusnutdinova and colleagues reported a platinum/copper complex capable of the cooperative C–H activation of terminal alkynes (Fig. 11).^72^ In order to demonstrate that proximal interactions with a Cu center modify the reactivity of Pt, they showed that the Cu/Pt bimetallic complex cleaved the C–H bond of 4-ethynylanisole while the Pt complex alone with the same ligand was completely inactive. The mechanistic study indicated that the Cu^I^ cationic center plays a crucial role in the coordination of the alkyne: it both allows the C–H bond to be placed near to the Pt^II^ center, while increasing its acidity, which then facilitates the oxidative addition to the Pt^II^ center and the formation of the hydride acetylide Pt^IV^. The reductive elimination of methane ultimately gives the acetylide complex, as shown on Fig. 11.

**Fig. 12:**
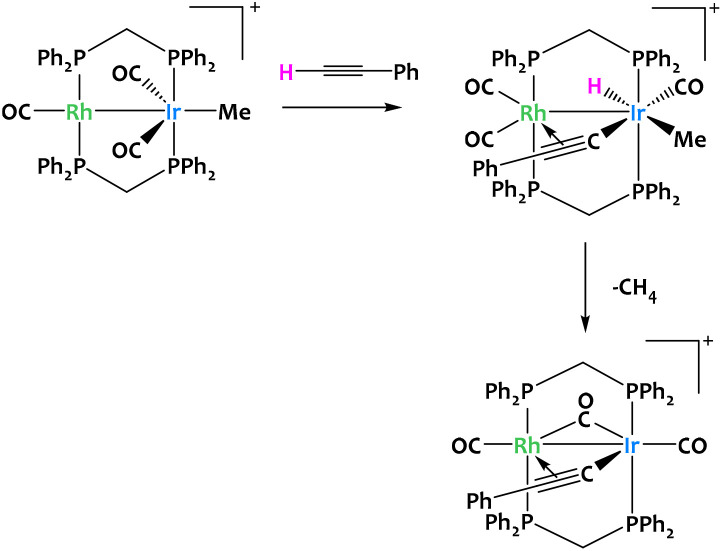
Cooperative C–H activation of terminal alkynes by a Rh/Ir complex.^73^

Such reactivity pattern seem prototypical of binuclear late transition metal systems, since a closely related alkyne activation was reported in 1996 for a Rh/Ir complex (Fig. 12).^73^

**Fig. 13:**
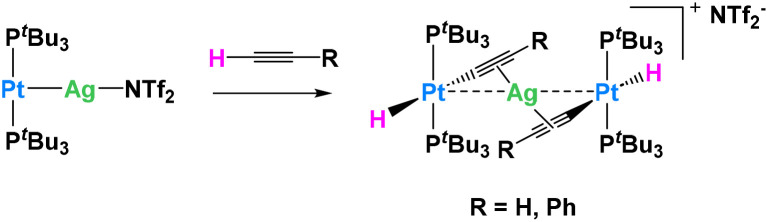
Cooperative C–H activation of terminal alkynes by a Ag/Pt Lewis pair.^74^

In another example, Maya, Campos and their collaborators explored the reactivity of Pt^0^/Ag^I^ metal-only Lewis pairs (MOLPs) in which the two metallic fragments are held together by a dative Pt → Ag bond (Fig. 13). They demonstrated that this system was capable of synergistically activating a wide variety of small molecules, including C–H bonds.^74^ This adduct was easily prepared in benzene or dichloromethane upon mixing [Pt(P^*t*^Bu_3_)_2_] and the silver salt Ag(NTf)_2_ in the absence of light. The platinum precursor [Pt(P^*t*^Bu_3_)_2_] by itself acts as a catalyst for C_2_H_2_ polymerization, causing the rapid precipitation of a dark purple solid upon exposure to the gas. The formation of the polymer is however inhibited in the presence of the silver salt, in which case the trinuclear complex, [(P^*t*^Bu_3_)_2_(H)Pt(μ-CCH)Ag(μ-CCH)Pt(H)(P^*t*^Bu_3_)_2_], is obtained quantitatively. The Pt⋯Ag distance is clearly lengthened (3.634(9) Å) compared to the MOLP (2.658(1) Å), indicating that the Pt → Ag dative bond is no longer present. A similar reactivity is derived from the addition of phenylacetylene (Fig. 13), resulting in the formation of the heterobimetallic dibridged bis-acetylide species, [(P^*t*^Bu_3_)_2_(H)Pt(μ-CCPh)Ag(μ-CCPh)Pt(H)(P^*t*^Bu_3_)_2_]. Importantly, none of the monometallic precursors ([Pt(P^*t*^Bu_3_)_2_] or Ag(NTf)_2_) show any reactivity with respect to phenylacetylene. The same group reported related Au(i)/Pt(0) MOFLPs featuring analogous reactivity.^75^

**Fig. 14:**
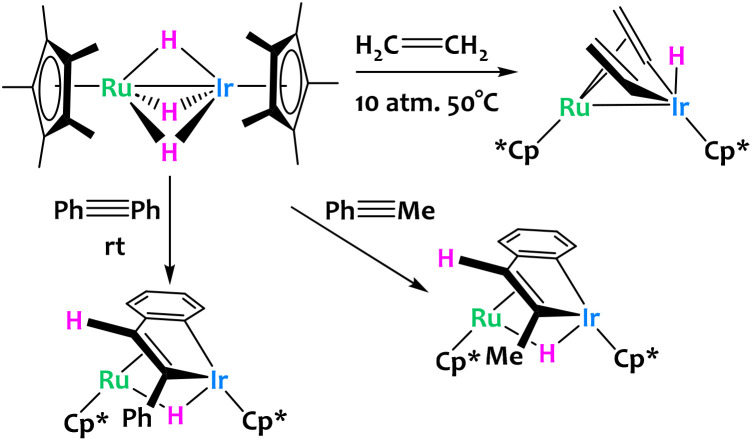
Ethylene and alkyne activation by a bimetallic Ru/Ir trihydride complex.^76^

Other examples of late/late heterobimetallic C–H activations come from Suzuki and coworkers, who reported a hydrido bridged Ru/Ir heterobimetallic complex which undergoes alkene and alkyne C–H activations (Fig. 14).^76^ When exposed to 10 atmospheres of ethylene, the system converts to the bis(μ-vinyl) complex in very high yields with concomitant formation of ethane from the direct hydrogenation of ethylene. The reaction of the same Ru/Ir complex with phenylacetylene derivatives is significantly different. Here, the complex inserts Ir and a hydride exclusively in the *trans* conformation with subsequent intramolecular activation of the *ortho* position of the phenyl ring to give the dinuclear benzoiridacyclopentadiene system shown on Fig. 14. A common theme of these C–H activation studies is the site selectivity of the complex; all C–H bond cleavages are reported to happen exclusively at the Ir center rather than Ru.

**Fig. 15:**
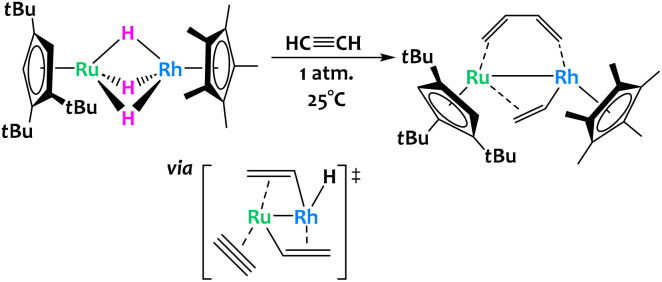
Cooperative C–H activation of acetylene with a Ru/Rh complex. The key transition state to the formation of the butadiene ligand occurs through acetylene insertion into a Ru–C bond.^77^

The activation of acetylene was reported across a related trihydrido bridged Ru/Rh bimetallic complex (Fig. 15).^77^ The authors found that exposure of this complex to one atmosphere of acetylene at room temperature allowed for its conversion into a μ-η^2^-vinyl-μ-η^2^:η^2^-*s-cis* butadiene complex in high yield. The mechanism for this remarkable conversion was difficult to ascertain through isotopic labelling or intermediate isolation and so the authors turned to computational studies. DFT showed that the critical transition state is the insertion of acetylene into a Ru–C bond of a bis(μ-vinyl) complex to give the butadiene core. Note that this insertion has been observed before for other homobimetallic Ru complexes.^78^

**Fig. 16:**
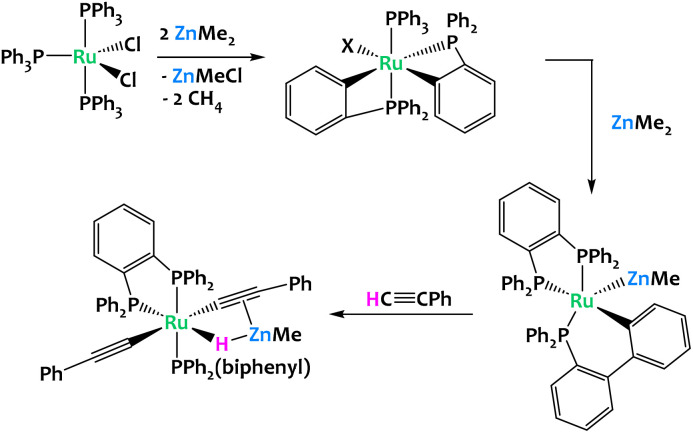
Multiple C–H activations promoted by a Ru/Zn system.^79^

Whittlesey and coworkers reported in 2019 the reaction of [Ru(PPh_3_)_3_Cl_2_] with excess ZnMe_2_, which led to multiple C–H bond activation of the aryl groups from the phosphine ligands, coupled with P–C and C–C bond formation and cleavage processes. This resulted in a chelating diphenylphosphinobenzene ligand as well as a cyclometallated (diphenylphosphino)biphenyl group in the final product of the reaction, [Ru(dppbz)(PPh_2_(biphenyl))(ZnMe)] (dppbz = 1,2-bis(diphenylphosphino)benzene); (Fig. 16). The resulting Ru/Zn heterobimetallic complex was shown to promote the cleavage of the C–H bond of phenylacetylene across the Ru–Zn bond.^79^

**Fig. 17:**
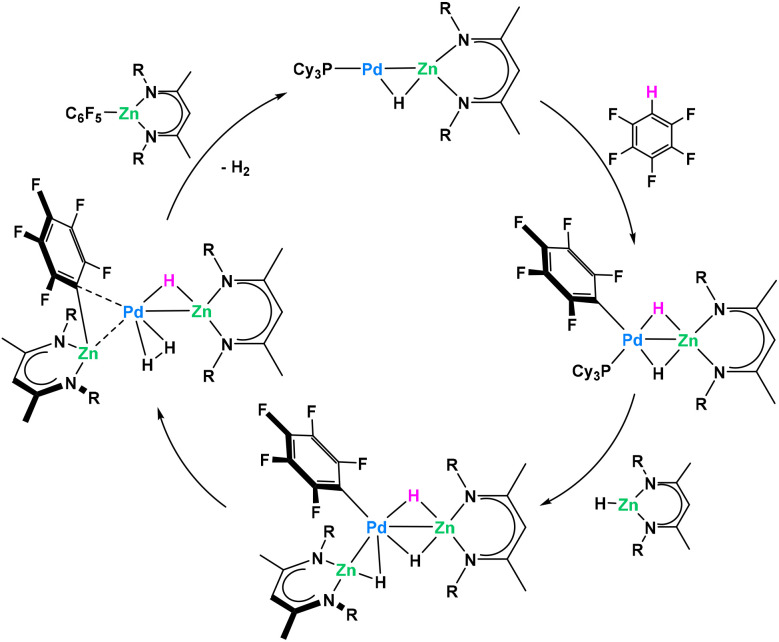
Pd/Zn heterometallic cooperativity in the C–H functionalization of fluoroarenes.^80^

Recently Crimmin reported a catalytic C–H functionalization by reaction of fluoro- or heteroarenes with a β-diketiminate supported Zn hydride in the presence of catalytic amounts of Pd(PCy_3_)_2_, to yield valuable organozinc compounds (Fig. 17).^80^ In order to investigate the mechanism, authors attempted the model reaction between stoichiometric amounts of the Zn hydride and Pd(PCy_3_)_2_. Due to equilibrium strongly favouring the starting materials, only the reversible binding between monometallic reagents was identified even at low temperature. Despite the problematic trapping of this putative Pd/Zn heterometallic intermediate, authors managed to isolate and characterize a direct Pt/Zn analogue stable at room temperature. This isolated intermediate follows the C–H activation proposed by the authors with rapid C–H activation of the fluoroarene. Control experiments with monometallic Pd(PCy_3_)_2_ and C_6_F_5_H only resulted in the trace formation of C–F activation product at Pd(0) center. Combined DFT and kinetic studies allowed proposing a catalytic cycle where heterobimetallic Pd/Zn species were shown to play the key role in the overall process (Fig. 17).

**Fig. 18:**
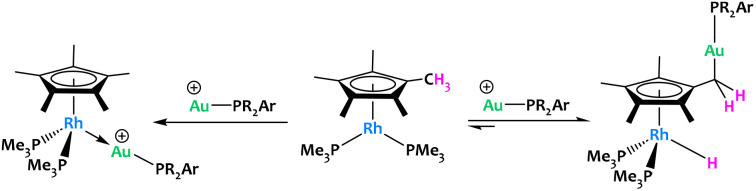
Reversible CH_3_ hydride migration to Rh promoted promoted by electrophilic gold fragments.^81,82^

Campos *et al.* reported an original example of cooperative C–H activation with a Rh/Au system (Fig. 18).^81^ Treatment of [Cp*Rh(PMe_3_)_2_] with electrophilic gold [(PR_2_Ar′)Au(NTf_2_)] complexes led to contrasting reactivities depending on the steric profile of the latter. MOLPs featuring a direct Rh → Au interaction were formed when the sterics of the Au(i) phosphine ligands were sufficiently small (Fig. 18-left). This stands in stark contrast to the most hindered gold species, which promoted an unprecedented, reversible hydride migration from a methyl group of C_5_Me_5_ to the rhodium(i) fragment (Fig. 18-right). The resulting Rh/Au species cooperatively cleaved polar E–H bonds (E = O, N), an unusual reactivity in which the methyl groups of the C_5_Me_5_ ligand acted as proton shuttles, which could offer new opportunities in catalysis in the future. A recent follow-up study from the same group has shown that the basicity of the phosphines bound to Rh also play an important role in these Cp* non-innocent behaviour.^82^

## 4. Early/early transition metal systems

When considering the field of heterobimetallic complexes, one notices a sincere scarcity of reports of early/early systems. Beyond the highly reactive nature of the early transition metals, early/early systems can also offer unusual coordination geometries and small molecule binding modes which may be critical for some interesting functionalization mechanisms. While most examples of early/early heterobimetallic complexes reported in the literature are used for olefin polymerization (with improved catalyst performance resulting from heterobimetallic effects),^83–87^ relatively few reports highlight clear early/early bimetallic cooperative C–H activation phenomena.

**Fig. 19:**
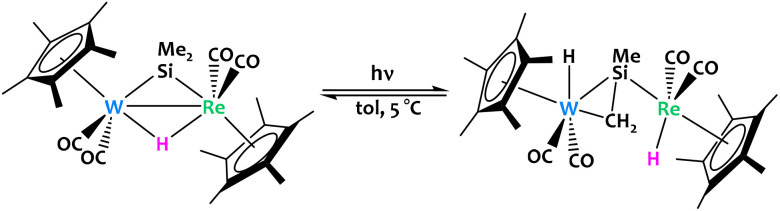
Reversible C–H activation was observed from a W/Re bimetallic complex under irradiation.^88^

Horino and coworkers reported a W/Re bimetallic complex featuring bridging hydride and dimethylsilylene ligands (Fig. 19).^88^ Photolysis partially converts the complex into two isomers of a μ-η^1^,η^2^-silenyl-bridged species *via* activation of a methyl C_sp^3^_–H bond. According to *in situ* NMR experiments, the reaction is reversible and the position of the chemical equilibrium strongly depends on the temperature.

**Fig. 20:**
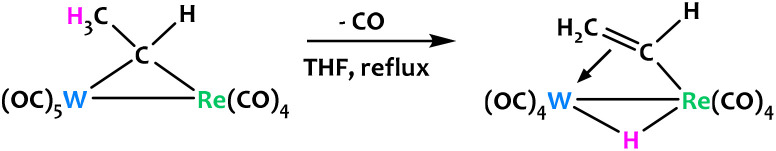
A W/Re μ-alkylidene undergoes intraligand C–H activation at high temperatures.^89^

Jeffery, Went and coworkers described a similar equilibrium between the μ-alkylidene ligand of an anionic W/Re heterobimetallic complex and its vinyl hydride isomer (Fig. 20).^89^ The [Re(CO)_4_(μ-CHMe)W(CO)_4_] species is stable at ambient temperature, but intraligand C–H activation is favoured when a solution of the complex in THF is refluxed. The isomerization at higher temperatures is accompanied by the loss of a carbonyl ligand on the W atom as a result of the π-coordination of the bridging vinyl ligand.

**Fig. 21:**
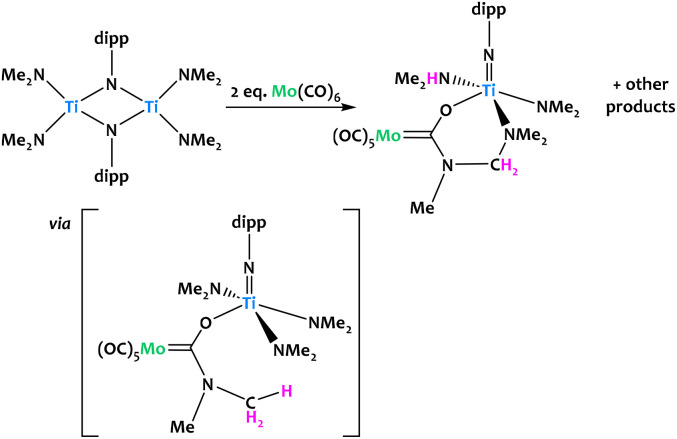
Titanium amido complexes rapidly react with Mo(CO)_6_ to give the Fischer carbene complex in a C–H activation step.^90^

Lorber and Vendier reported the unexpected C–H activation of a dimethylamido ligand on an imido-bridged titanium bimetallic complex upon the addition of Mo(CO)_6_ (Fig. 21).^90^ The authors propose that the formation of the Fischer aminocarbene is likely through the nucleophilic attack of NMe_2_ at the carbon atom of a coordinated CO ligand on Mo. This would generate the titanoxy-aminocarbene intermediate which undergoes a subsequent C–H activation, likely through the loss of a proton, promoted by another dimethylamido fragment. C–N coupling with a further dimethylamido unit was confirmed from single-crystal X-ray diffraction. Several other unidentified products were observed, which given the stoichiometry of the reaction, was not unexpected.

**Fig. 22:**
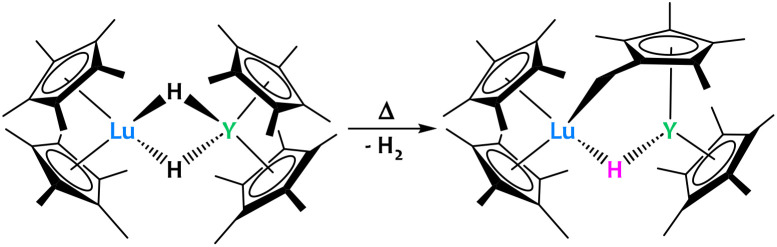
The formation of a Lu/Y “tucked-over” system occurs when heating the bridged hydrido complex.^91^

Lastly, Evans and Furche reported a Lu/Y dihydrido heterobimetallic complex which, when heated, activates a methyl group on the Cp* ring to give a “tucked over” complex (Fig. 22).^91^ Two products were observed from this reaction: the one presented above with η^1^ coordination of the “tucked in” Cp* to Lu and the opposite, with the η^1^ coordination to Y. Nevertheless, the advantage of having a heterobimetallic system was not proven in this particular case since homobimetallic systems featured similar reactivity.^91–93^

## 5. Heterobimetallic systems involving main-group metallic elements

Main-group Lewis acids have often been used as external partners in a number of catalytic processes involving transition metals. In the field of C–H activation, main group additives have been seen to drive the selectivity at specific positions on Lewis basic substrates.^94–101^ More recently, transition metal complexes with main group metalloligands have attracted increased interest in the hope of achieving unusual cooperative reactivity. As the examples below illustrate, d-block/aluminium compounds have proved particularly successful in this respect.^35,37,62,102,103^

**Fig. 23:**
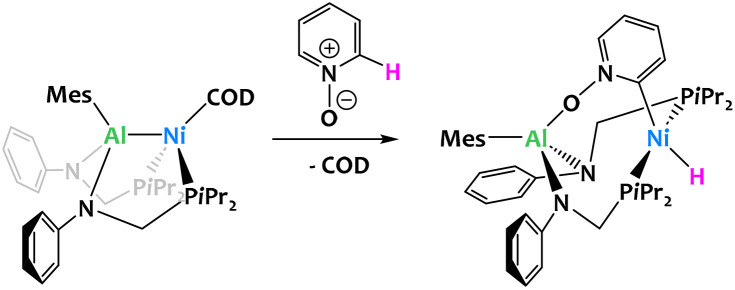
Al-directed *ortho* C–H oxidative addition of pyridine N-oxide by an aluminium–nickel complex.^104^

Lu and coworkers reported an aluminium–nickel complex which reacts with pyridine *N*-oxide to give a C–H activation product shown in Fig. 23. In this example, the aluminium ligand serves to orientate the selectivity at the *ortho* position, but the reaction mechanism is proposed to take place through a classical oxidative addition at the nickel center.

**Fig. 24:**
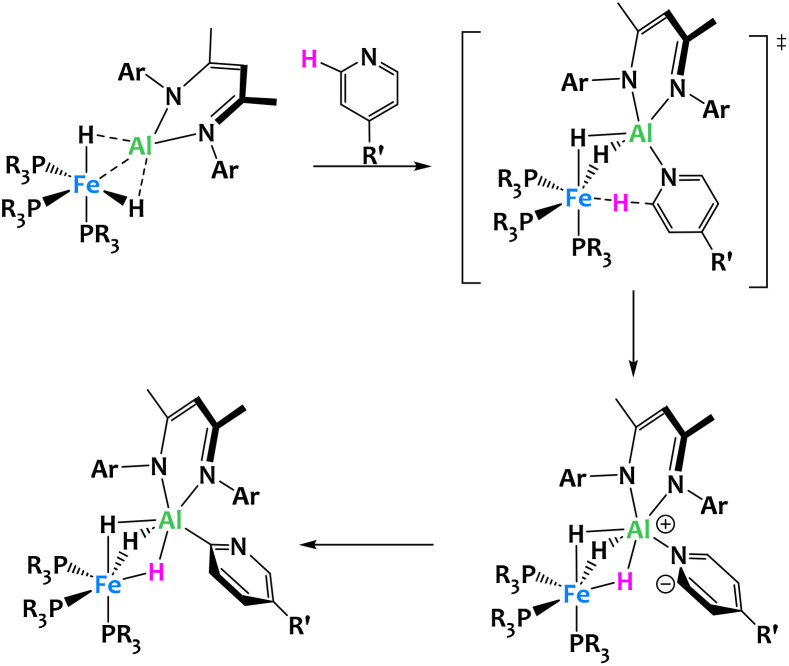
Cooperative C–H activation of pyridine by a Fe/Al complex.^105,106^

Crimmin and coworkers recently described the inclusion of an electropositive aluminium-based ligand in the octahedral coordination sphere of an iron hydrido complex, leading to unexpected reactivity (Fig. 24). The resulting Fe/Al heterobimetallic complex was found to selectively activate the C–H bonds in the *ortho* position of pyridine derivatives with electron-donating substituents in the *para* position (R′ = Me, Ph and NMe_2_). Mechanistic studies on the reactivity of this complex suggest that the *ortho* C–H activation of pyridine occurs in two steps. The first is the deprotonation of the pyridine substrate following the coordination of the nitrogen to the aluminium center. The second step is the switch from N- to C-coordination of the deprotonated pyridine and formation of the Al–C bond. Within this complex, the Fe–Al bond is highly polarized. The destabilized HOMO, localized predominantly on Fe, acts as a Lewis donor orbital, and the empty p orbitals of the LUMO+6, on the Al center, serve as Lewis acceptors. Note that related boryl-metal cooperative activation of the C–H bonds of pyridines have also been reported.^107–109^

**Fig. 25:**
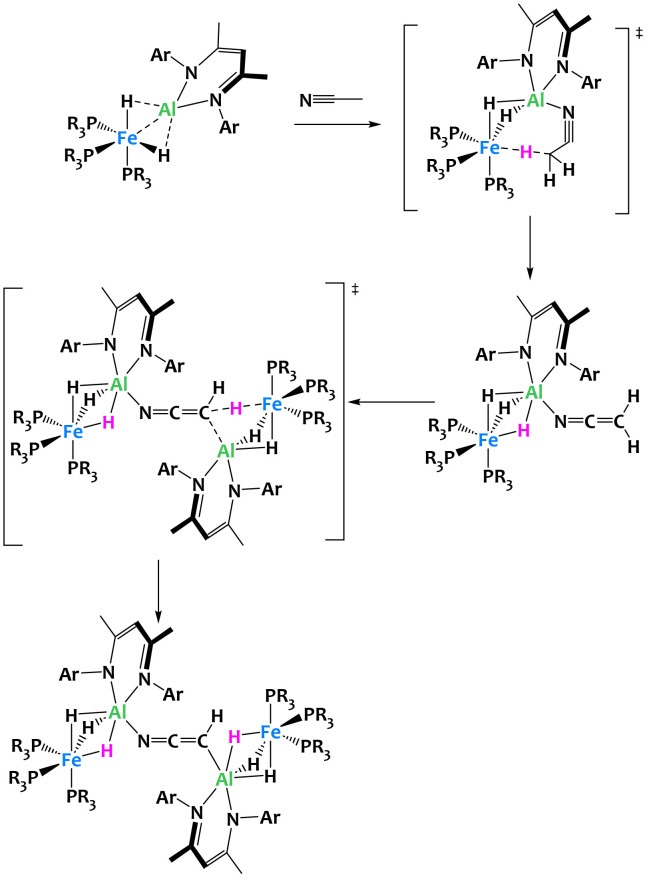
Cooperative C–H activation of acetonitrile by a Fe/Al complex.^110^

Further investigations into this Fe/Al bimetallic complex by the same group confirmed its extraordinary basicity, resulting in the rare ability to perform a double deprotonation of acetonitrile. The reaction of CH_3_CN with two equivalents of the binuclear complex yields a coordination compound in which two Fe/Al subunits are bridged by a μ_2_-κ_C_,κ_N_-[CHCN]^2−^ dianion (Fig. 25).^110^*In situ* characterization of the reaction reveals that the double deprotonation in fact occurs stepwise. First, a rapid deprotonation by one equivalent of the bimetallic species leads to an Al ketene imide complex which subsequently transfers a second proton from the N-coordinated α-nitrile anion to the second equivalent of the Fe/Al complex. DFT calculations corroborate this reaction mechanism and emphasize that the pre-coordination to Al is crucial in both deprotonation steps by increasing the negative charge localization on Fe and by facilitating a favourable spatial arrangement for the proton transfer. This example highlights once again the importance of metal–metal cooperativity to access novel reactivity not previously achieved with monometallic complexes.

**Fig. 26:**
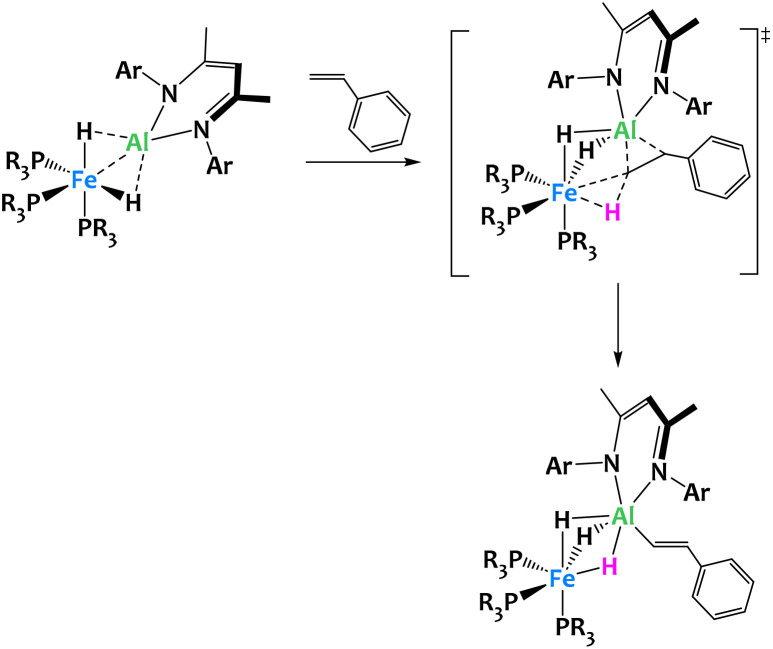
Cooperative C–H activation of vinyl derivatives by a Fe/Al complex.^111^

The same system enables the selective vinylic C–H activation of styrene derivatives, giving only the (*E*)-β-metalation products (Fig. 26).^111^ The reaction pathway involves the formation of a pseudo [2 + 2] cycloadduct from the reaction of the alkene with the metal–metal bond. This cycloaddition intermediate is then converted into the hydrido vinyl product in a rate-determining C–H cleavage step, a highly unusual oxidative addition of a non-aromatic C_(sp^2^)_–H bond across two adjacent metal centers. The transition state for this step is asynchronous and features characteristics of both a concerted and a stepwise mechanism. Here, metal–metal interactions and the spatial proximity between Fe and Al play a special role in two ways. First, unlike in the case of monometallic systems, the alkene coordination to the metal centers of the bimetallic complex is reversible and does not compete with C–H activation but is in fact an essential prerequisite initiating the bond cleavage. Secondly, the regioselectivity and stereospecificity of the reaction directly arise from the decisive last transition state and therefore require the participation of both metal centers.^111^

**Fig. 27:**
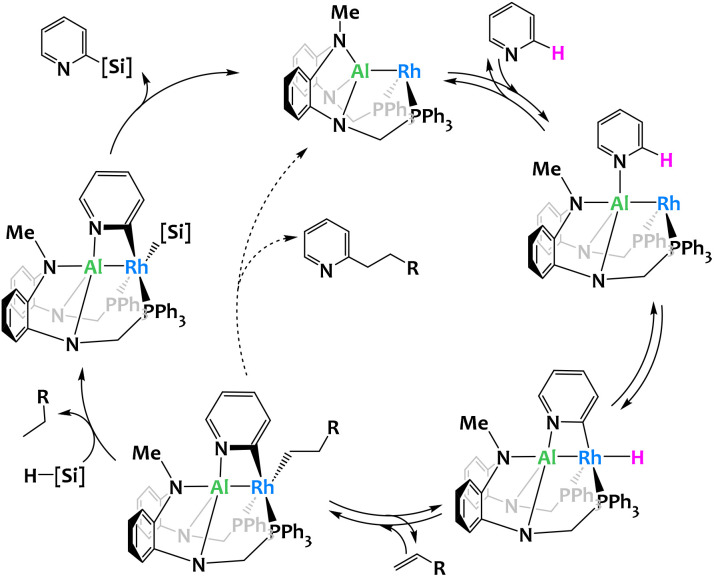
Proposed mechanism for the selective C2-silylation or alkylation of pyridines catalyzed by a Rh/Al complex.^112,113^

While the previous examples are stoichiometric activations, the group of Nakao reported a Rh–Al complex^114^ able to catalyze the C2-selective functionalization (silylation or alkylation) of pyridines.^112,113,115^ The Rh center is coordinated by an X-type PAlP pincer ligand and provides a catalytic site for the regioselective oxidative insertion into the C2–H bond of pyridine. The ensuing hydrometallation of the alkene leads to an intermediate species which, depending on reaction conditions and the targeted product, either generates the 2-alkylated pyridine *via* reductive elimination or, in the presence of a hydrosilane, gives rise to the formation of the corresponding alkane and a 2-pyridyl-silyl complex. Reductive elimination from the latter gives the 2-silylpyridine, a valuable organic building block difficult to access by conventional, monometallic catalysis (Fig. 27). The 2-pyridyl-silyl intermediate is isolable and proves that the C–Si bond formation occurs at the Rh site while the distal Al center assists by coordinating the Lewis-basic nitrogen atom, thus holding the pyridine molecule in place and preventing obstruction of the proximal catalytic site through the N-coordination commonly encountered with monometallic, late transition-metal catalysts. Deuterium-labeling and computational studies indicate that all elementary steps except the rate-determining reductive elimination are reversible and benefit from the proposed Rh/Al cooperation. These results further stress the importance of the catalyst's bimetallic nature.

**Fig. 28:**
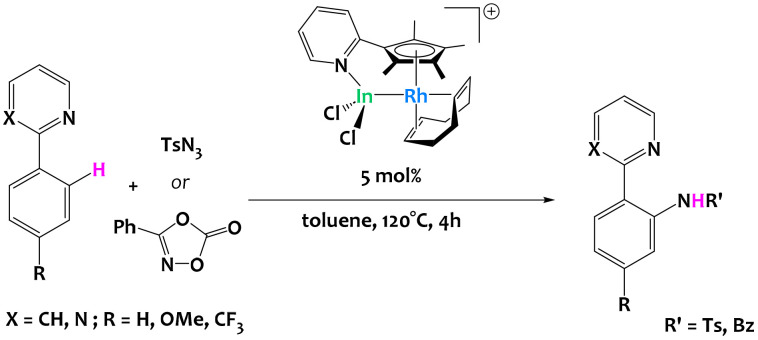
Rhodium-catalyzed C–H activation of 2-arylpyridines enabled by an indium metalloligand.^116^

Takaya and coworkers reported the use of a pyridine-tethered cyclopentadienyl ligand to prepare a heterobimetallic Rh/In complex featuring a metal–metal bond (Fig. 28). The Z-type In metalloligand changes the electronic environment and redox properties of the rhodium metal, which leads to catalytic activity in sp^2^ C–H bond activation and functionalization of various 2-arylpyridines. Importantly, the monometallic complexes Cp*Rh(COD) and [Cp*RhCl]_2_ did not catalyze the reaction and analogous Al and Ga systems resulted in poor C–H activation catalytic performances, demonstrating the interest of In as a metalloligand.^116^

**Fig. 29:**
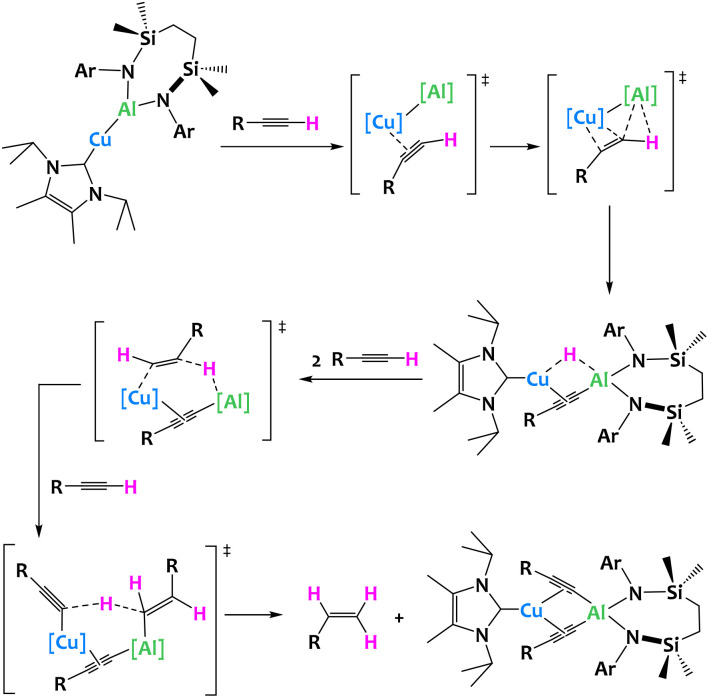
Cooperative C–H activation of terminal acetylenes by a Cu/Al complex.^117^

Very recently, a unique terminal alkyne transformation, mediated by a copper(i) alumanyl derivative was described by McMullin, Hill and coworkers. This heterobimetallic complex promotes alkyne transfer semi-hydrogenation in which the acidic alkyne itself acts as the proton source. Through the isolation of several reaction intermediates, in combination with computational studies, the authors have shown that a novel mechanism is at play, where the Cu–Al σ bond first activates the C–H acidic alkyne bond, leading to a heterobimetallic hydride (Fig. 29).^117^

**Fig. 30:**
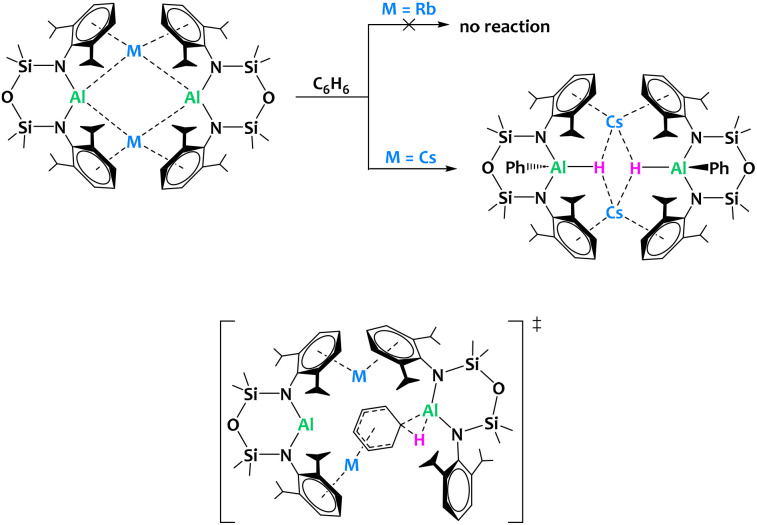
Alkali metal facilitated aromatic C–H bond activation by an aluminyl species (top) and proposed structure for the transition state according to DFT calculations (bottom).^118^

McMullin, Coles, Mulvey and coworkers reported the preparation of alkali aluminyls of general formula [M{Al(NON^Dipp^)}]_2_ (M = Li–Cs) (Fig. 30). Interestingly, despite their structural similarity, the Cs derivative is the only compound in the series enabling the C–H bond activation of benzene leading to oxidative addition onto the Al(i) center, pointing towards a significant alkali metal/aluminium synergistic effect. The barrier to oxidative addition of the C–H bond onto the aluminyl center computed by DFT is higher for Rb than Cs (28.3 *vs.* 23.7 kcal mol^−1^), which is compatible with the experimental observation. A negatively charged Meisenheimer intermediate, stabilized by the coordination of the alkali cation to the activated arene, precedes the C–H activation state (Fig. 30). This example illustrates that subtle changes in the nature of the metal/metal combination can have drastic impact on the reactivity outcome.

**Fig. 31:**
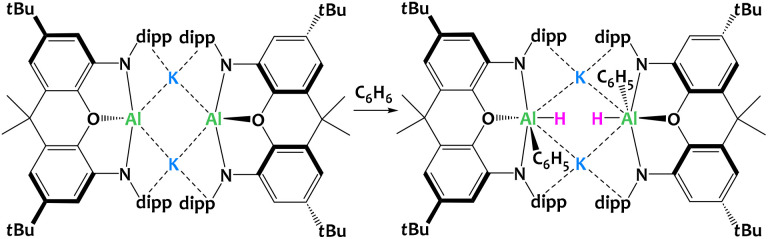
Formal oxidative addition of the C–H bond in benzene at the Al^I^ centres in dimethylxanthene-stabilized potassium aluminyl complex. Dipp = diisopropylphenyl.^119,120^

Goicoechea, Aldridge and collaborators described a related aluminyl system (Fig. 31) where the ligand is a sterically demanding dimethylxanthene derivative.^119,120^ Contrary to the system reported by McMullin, Coles and Mulvey (Fig. 30), here the potassium aluminyl [K{Al(NON)}]_2_ dimer is able to cleave sp^2^ C–H bonds *via* formal oxidative addition to the Al^I^ center. The reactivity of the system has also been illustrated with substituted aromatics such as toluene, xylenes and other alkylbenzenes, producing a mixture of species arising from competitive benzylic C–H bond cleavage or the activation of *meta*-C_sp^2^_–H. The potassium cations hold the dimer through cation–π interactions with the Dipp moieties, and presumably play a key role in this S_N_Ar-like aluminyl reactivity akin to that described above. Indeed, when the potassium cations are encapsulated by 2.2.2-cryptand, the selectivity changes and oxidative addition of the C–C bond in benzene is observed in place of the C–H activation.^121^

**Fig. 32:**
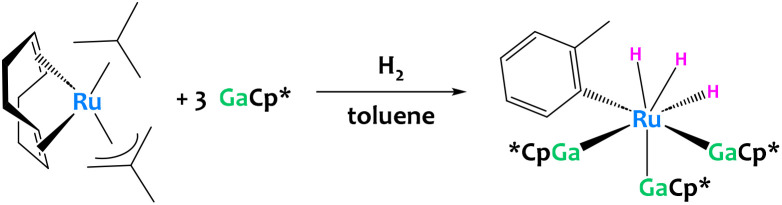
C–H activation of toluene promoted by a low-valent Ru/Ga system. The structure of the resulting aryl complex was inferred from mass spectrometry and DFT calculations.^122^

C–H activation of toluene was also reported recently by a low-valent Ru/Ga system (Fig. 32). Unfortunately the aryl complex produced is too unstable for isolation, preventing the determination of its definitive structure and the elucidation of the reaction mechanisms.^122^

**Fig. 33:**
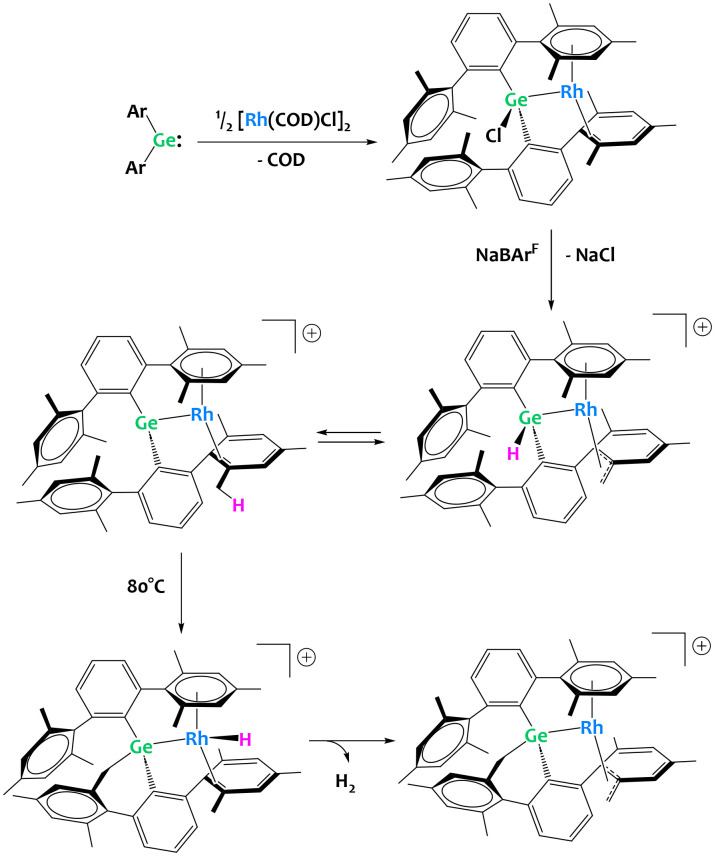
Multiple C–H activation events at a rhodium germylene complex.^123^

Group 14 metalloids are also gaining attention as competent partners in heterobimetallic C–H activation complexes. The Campos group has made forays into transition metal/p-block heterobimetallic systems with their recent report of a “naked” Rh(i) germylene complex (Fig. 33).^123^ In this example, chloride abstraction yields a cationic germylene fragment capable of double C–H bond activation with the benzylic positions of the ligand. Here, the cooperativity between Ge and Rh is on display, as the computationally derived mechanism clearly exhibits the interplay between Rh and Ge with a detailed discussion on how the metal–metal bonding changes character during the reaction progress.^123^

**Fig. 34:**
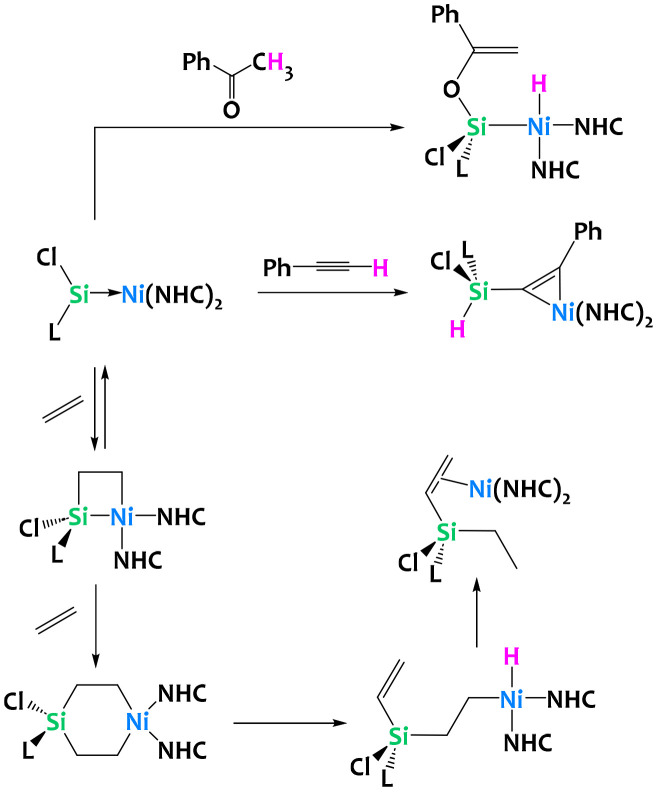
C–H activation chemistry of a silylene–nickel(0) complex with acetophenone, phenyl acetylene and ethylene.^124^

Driess and coworkers have recently reported cycloadditions of unsaturated substrates to a Ni(0) silylene complex (Fig. 34). Addition of acetophenone generated a Ni(ii) hydride after deprotonation of the [2 + 2] cycloaddition product and enolization. The addition of phenylacetylene however yields a Ni(0) π-complex with C–H activation at the Si(ii) silyene. Perhaps more relevant, the reversible ethylene addition to the Ni(0) silylene gives the formal [2 + 2] nickelasila-cyclobutane. Computational analysis of the reaction mechanism suggests this reaction operates through an initial [2 + 1] η^2^-adduct with spontaneous ring-expansion to give the metallasila-cyclobutane. Addition of another equivalent of ethylene yields, after sequential β-hydride elimination and reductive elimination, a silylated alkene Ni(0) π-complex (Fig. 34). Analogous reactivity is observed with other alkenes, most notably styrene.^124^

These recent examples show that the p-block offers plenty of opportunities. Bimetallic complexes built from s-block elements have also led to very efficient cooperative C–H activations through unique mechanisms.

**Fig. 35:**
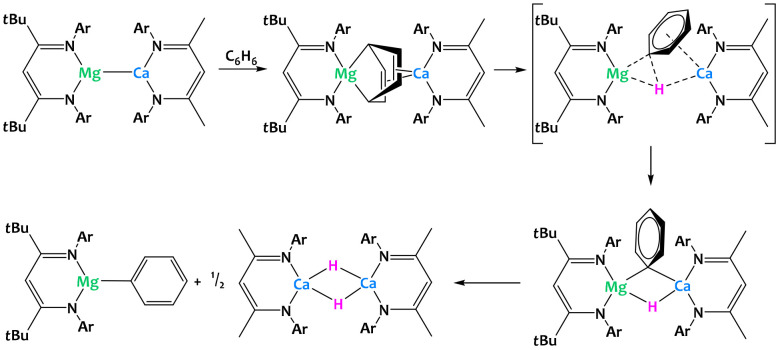
Cooperative C–H activation of benzene promoted by a calcium–magnesium complex.^125^

Harder and coworkers recently described a low-valent group 2 heterobimetallic complex with magnesium and calcium (Fig. 35).^125^ Despite attempts to sterically stabilize the complex using bulky β-diketiminate ligands, the species is only proposed as a short-lived intermediate but has not been isolated and structurally characterized. Obtained through salt metathesis between the corresponding sodium magnesyl and calcium iodide precursors, the proposed complex directly reduces a solvent molecule, yielding a Mg(μ_2_,μ_4_-C_6_H_6_)Ca-bridged dinuclear complex. DFT calculations suggest that this step is followed by intramolecular C–H bond cleavage and formation of the monometallic phenylmagnesium and calcium hydride complexes, the latter of which crystallizes as a dimer from THF. The overall result is the activation of a benzene C–H bond *via* cooperative reduction to a bridging C_6_H_6_^2−^ dianion and spatial separation of the phenyl and the hydride moieties as parts of two distinct molecules.^125^

**Fig. 36:**
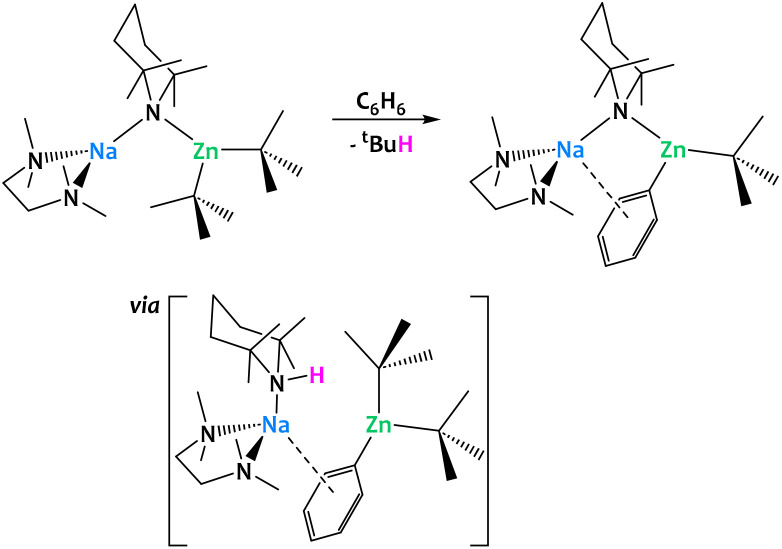
Alkali–metal-mediated metalations (AMMMs)^126,127^ developed by Mulvey and co-workers: TMP-mediated cooperation with the alkali–metal ion allows for the zincation of aromatic C–H bonds.

More generally, alkali–metal reagents have been shown to be powerful additives in a great number of dehydrometalations, enabling chemical transformations that could not be accomplished by a single, less electropositive metal center. Mulvey and coworkers developed a series of alkali–metal-mediated metalations (AMMMs),^126,127^ among them magnesiations (AMMMg),^128^ zincations (AMMZn)^129^ and manganations (AMMMn^II^),^130,131^ which rely on the formation of a heterobimetallic alkyl amide of the general composition [(TMEDA)A^I^(μ-TMP)(μ-R)M^II^(R,TMP)] (A^I^ = Li, Na; M^II^ = Mg^II^, Zn^II^, Mn^II^; R = ^*n*^Bu, ^*t*^Bu, CH_2_SiMe_3_; TMEDA = *N*,*N*,*N*′,*N*′-tetramethylethylenediamine, TMP = 2,2,6,6-tetramethylpiperidine) (Fig. 36). These species display a remarkable reactivity towards the C_sp^2^_−H bonds of benzene, and other (hetero-)aromatic compounds, to perform C–H activations that cannot be realized by their monometallic counterparts, *i.e.* without assistance provided by an alkali–metal ion. In all three cases, this increased reactivity essentially stems from two common features: the permanent spatial proximity between the alkali and the divalent metal centers enforced by the bridging N and/or C-donor bases and the σ/π cooperation between the two metal ions. Single-crystal X-ray crystallography unambiguously confirms the presence of a phenyl ligand σ-bound to Mg/Zn/Mn as well as a π contact to the more distant Na ion. Complementary DFT calculations of the AMMZn reaction conducted by Nobuto and Uchiyama indicate that the dehydrometalation most likely proceeds in two steps.^132^ Instead of a direct substitution of an alkyl ligand by the phenyl moiety, the amido ligand first acts as the kinetic base deprotonating benzene to afford the monoaryl–bisalkyl intermediate [(TMEDA)·Na(μ-R)(μ-Ph)Zn(R)] with concomitant formation of TMP(H). In a second acid–base reaction, TMP(H) attacks the bridging alkyl ligand to release the final product and the alkane. An excess of the heterobimetallic complex can lead to more complex architectures through multiple, iterative substitutions on the same substrate. Among the products formed are notably inverse-crown complexes,^133^ which have been extensively reviewed elsewhere.^126,127^

**Fig. 37:**
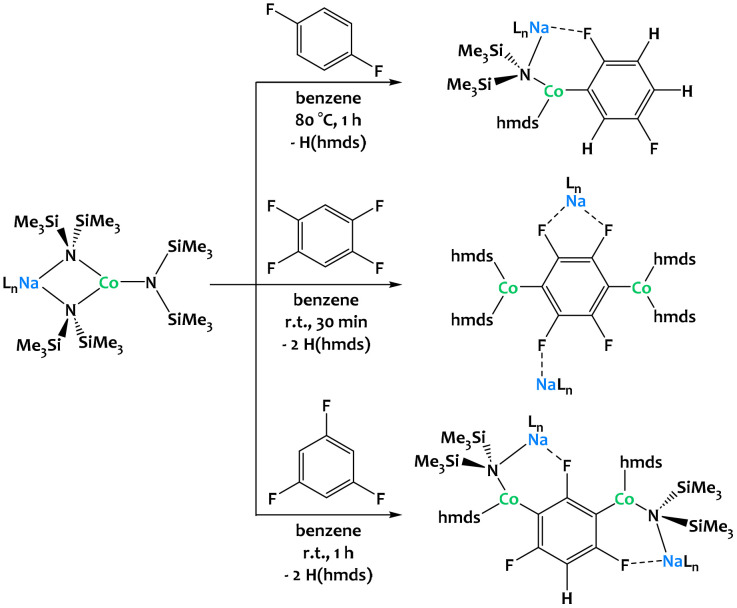
Regioselective multi-functionalization of fluoroarenes *via* C–H activation mediated by alkali–Co couples.^134,135^

Hevia and coworkers have considerably extended the alkali–metal -*ate* chemistry^136–138^ and very recently reported the dehydrometallation of fluoroarenes mediated by cobalt alkali–metal amides (Fig. 37).^134,135^ Regioselective functionalization of fluoroarenes can be challenging due to uncontrolled reactivity and unwanted side reactions such as benzyne formation or cascade processes (auto-metalation, multi-metalation).^138^ Heterobimetallic amide bases combining an alkali metal with a divalent transition-metal center not only prevent these side reactions but also allow new reaction pathways towards thermally stable metalates under mild conditions. Unlike in many established C–H bond activation processes, the oxidation state of the 3d-transition metal remains unchanged throughout the reaction. In contrast to Mulvey's AMMMs, the alkali–metal ions partake in the C–H activation and direct the metalation through intramolecular coordination to the fluorine atoms. The importance of these interactions is further underlined by the fact that the choice of the alkali metal matters, as showcased by considerable differences in reactivity when descending from Li through Na to K.^135^ While the reaction of 1,3,5-trifluorobenzene with three equivalents of [NaCo(hmds)_3_] (Fig. 37, bottom) promotes 1,3-dicobaltation, reactions with the same amounts of [LiCo(hmds)_3_] and [KCo(hmds)_3_] only lead to monocobaltation or no reaction at all, respectively, even under forcing reaction conditions (16 h, 80 °C). Similar reactivity can be observed with related Fe and Zn complexes.^139–141^

## 6. Conclusion and perspectives

Over the past years, a diverse array of heterobimetallic systems for cooperative activation of C–H bonds has been uncovered. Although the number of well characterized examples is so far limited to relatively few architectures, they clearly illustrate the enormous possibilities of this emerging field. However, *catalytic* functionalization of hydrocarbons beyond the first C–H activation is far more difficult to achieve. Many of the examples described above utilize activated C–H bonds, such as in terminal alkyne derivatives; work dedicated to the activation of unactivated aliphatic C–H bonds is still needed. Ultimately, the selective activation of specific C–H bonds in complex organic structures, with robust functional group tolerance, is an important milestone to be addressed.

To reach these goals, the clever design of new organometallic systems is needed. The challenge is to prepare compounds in which the two metals are at close proximity to allow for bimetallic cooperativity, and which are sufficiently robust to keep these assemblies intact during the course of catalytic processes. With this in mind, and as seen from the examples listed in this Perspective, either direct M–M′ bonds or with a number of bridging hydrides between metals offers excellent opportunity for minimizing steric encumbrance around the metal centers and ensuring a substrate can access both metals simultaneously for cooperative substrate activation.

The choice of metal partners is, of course, an essential aspect of heterobimetallic complexes. With an entire periodic table to choose from, one could imagine a near-infinite combination of elements. Promising results stem from the inherently polarized bonding of early/late combination of elements, although late/late and transition/main-group metal combinations have all also produced impressive results. Heterotrimetallic species, and systems of even higher nuclearities also provide an avenue for further exploration.

The nature of supporting ligands around both metals can also play a critical role. Multidentate ligand platforms can bring metals within proximity (as seen in Fig. 11, 12, 23 and 27), but attention must be given not to saturate the metals’ coordination spheres. Furthermore, the architectural support given by the ligand can also extend to directly participating in the reactivity of the complex *a la* metal ligand cooperativity. One recent report by Mankad and coworkers highlights an unprecedented radical pair pathway for the activation of oxygenated substrates.^37^ The authors postulate that the Al–Fe bond dissociates homolytically to generate formally Al^II^ and Fe^I^ metalloradicals, responsible for the surprising reactivity. Such mechanism has yet to be demonstrated for C–H bonds activation, but could open new possibilities and considerations when designing novel bimetallic systems.

We hope that this contribution will inspire chemists to further look at promising metal–metal cooperative reactivities for catalytic C–H bond functionalization and stimulate progress in the future. As with many fields in inorganic chemistry, the problem today is how to design transition metal complexes. With a keen eye on the design features discussed above, we believe that catalytic functionalization of unactivated C–H bonds is on the horizon. Ultimately, the fundamental mechanistic knowledge gained from well-designed and well-explored heterobimetallic systems will extend far beyond homogeneous catalysis. Results and mechanisms from homogeneous cases have already seen practical applications in bimetallic heterogeneous catalysis.^60,142,143^

## Authors information

The authors wish it to be known that, in their opinion, the four first authors should be regarded as joint first authors. The order of co-first authorship was determined by alphabetical order. Co-first authors can prioritize their names when adding this article to their respective résumés.

## Conflicts of interest

There are no conflicts of interest to declare.

## Supplementary Material

## References

[cit1] Andino J. G., Kilgore U. J., Pink M., Ozarowski A., Krzystek J., Telser J., Baik M. H., Mindiola D. J. (2010). Chem. Sci..

[cit2] Zhang S., Tamm M., Nomura K. (2011). Organometallics.

[cit3] Fostvedt J. I., Grant L. N., Kriegel B. M., Obenhuber A. H., Lohrey T. D., Bergman R. G., Arnold J. (2020). Chem. Sci..

[cit4] Flores J. A., Cavaliere V. N., Buck D., Pintér B., Chen G., Crestani M. G., Baik M. H., Mindiola D. J. (2011). Chem. Sci..

[cit5] Bailey B. C., Fan H., Baum E. W., Huffman J. C., Baik M. H., Mindiola D. J. (2005). J. Am. Chem. Soc..

[cit6] Bailey B. C., Fan H., Huffman J. C., Baik M. H., Mindiola D. J. (2007). J. Am. Chem. Soc..

[cit7] Vilardo J. S., Lockwood M. A., Hanson L. G., Clark J. R., Parkin B. C., Fanwick P. E., Rothwell I. P. (1997). J. Chem. Soc., Dalton Trans..

[cit8] Wolczanski P. T. (2018). Organometallics.

[cit9] Carbó J. J., García-López D., Gómez-Pantoja M., González-Pérez J. I., Martín A., Mena M., Santamaría C. (2017). Organometallics.

[cit10] Walsh P. J., Hollander F. J., Bergman R. G. (1988). J. Am. Chem. Soc..

[cit11] Cummins C. C., Baxter S. M., Wolczanski P. T. (1988). J. Am. Chem. Soc..

[cit12] Ackermann L., Vicente R., Althammer A. (2008). Org. Lett..

[cit13] Özdemir I., Demir S., Çetinkaya B., Gourlaouen C., Maseras F., Bruneau C., Dixneuf P. H. (2008). J. Am. Chem. Soc..

[cit14] Gorelsky S. I., Lapointe D., Fagnou K. (2008). J. Am. Chem. Soc..

[cit15] Ess D. H., Bischof S. M., Oxgaard J., Periana R. A., Goddard W. A. (2008). Organometallics.

[cit16] Davies D. L., Macgregor S. A., McMullin C. L. (2017). Chem. Rev..

[cit17] Altus K. M., Love J. A. (2021). Commun. Chem..

[cit18] Munz D., Meyer D., Strassner T. (2013). Organometallics.

[cit19] Wischert R., Copéret C., Delbecq F., Sautet P. (2011). Angew. Chem., Int. Ed..

[cit20] Joubert J., Salameh A., Krakoviack V., Delbecq F., Sautet P., Copéret C., Basset J. M. (2006). J. Phys. Chem. B.

[cit21] Conley M. P., Delley M. F., Núnez-Zarur F., Comas-Vives A., Copéret C. (2015). Inorg. Chem..

[cit22] Estes D. P., Siddiqi G., Allouche F., Kovtunov K. V., Safonova O. V., Trigub A. L., Koptyug I. V., Copéret C. (2016). J. Am. Chem. Soc..

[cit23] Fickenscher Z., Hey-Hawkins E. (2023). Molecules.

[cit24] Mankad N. P. (2016). Chem. – Eur. J..

[cit25] York J. T., Young V. G., Tolman W. B. (2006). Inorg. Chem..

[cit26] Park Y. J., Ziller J. W., Borovik A. S. (2011). J. Am. Chem. Soc..

[cit27] DeRosha D. E., Mercado B. Q., Lukat-Rodgers G., Rodgers K. R., Holland P. L. (2017). Angew. Chem., Int. Ed..

[cit28] Zhang H., Hatzis G. P., Moore C. E., Dickie D. A., Bezpalko M. W., Foxman B. M., Thomas C. M. (2019). J. Am. Chem. Soc..

[cit29] Charles R. M., Brewster T. P. (2021). Coord. Chem. Rev..

[cit30] Li Y., Su P., Jiang J., Ke Z. (2021). ACS Catal..

[cit31] Gramigna K. M., Dickie D. A., Foxman B. M., Thomas C. M. (2019). ACS Catal..

[cit32] Charles R. M., Yokley T. W., Schley N. D., Deyonker N. J., Brewster T. P. (2019). Inorg. Chem..

[cit33] Cammarota R. C., Lu C. C. (2015). J. Am. Chem. Soc..

[cit34] Karunananda M. K., Mankad N. P. (2015). J. Am. Chem. Soc..

[cit35] Escomel L., Del Rosal I., Maron L., Jeanneau E., Veyre L., Thieuleux C., Camp C. (2021). J. Am. Chem. Soc..

[cit36] Cooper O., Camp C., Pécaut J., Kefalidis C. E., Maron L., Gambarelli S., Mazzanti M. (2014). J. Am. Chem. Soc..

[cit37] Sinhababu S., Radzhabov M. R., Telser J., Mankad N. P. (2022). J. Am. Chem. Soc..

[cit38] Krogman J. P., Foxman B. M., Thomas C. M. (2011). J. Am. Chem. Soc..

[cit39] Bagherzadeh S., Mankad N. P. (2015). J. Am. Chem. Soc..

[cit40] Corona H., Pérez-Jiménez M., de la Cruz-Martínez F., Fernández I., Campos J. (2022). Angew. Chem..

[cit41] Sinhababu S., Lakliang Y., Mankad N. P. (2022). Dalton Trans..

[cit42] Powers I. G., Uyeda C. (2017). ACS Catal..

[cit43] Bodio E. , Picquet M. and Le Gendre P., in Topics in Organometallic Chemistry, Springer, Cham, 2015, vol. 59, pp. 139–186

[cit44] Batuecas M., Gorgas N., Crimmin M. R. (2021). Chem. Sci..

[cit45] Ritleng V., Chetcuti M. J. (2007). Chem. Rev..

[cit46] Cooper B. G., Napoline J. W., Thomas C. M. (2012). Catal. Rev..

[cit47] Gade L. H. (2000). Angew. Chem., Int. Ed..

[cit48] Baranger A. M., Bergman R. G. (1994). J. Am. Chem. Soc..

[cit49] Baranger A. M., Hollander F. J., Bergman R. G. (1993). J. Am. Chem. Soc..

[cit50] Hanna T. A., Baranger A. M., Bergman R. G. (1995). J. Am. Chem. Soc..

[cit51] Hanna T. A., Baranger A. M., Bergman R. G. (1996). Angew. Chem., Int. Ed. Engl..

[cit52] Fulton J. R., Hanna T. A., Bergman R. G. (2000). Organometallics.

[cit53] Pregosin P. S., Togni A., Venanzi L. M. (1981). Angew. Chem., Int. Ed. Engl..

[cit54] Howarth O. W., McAteer C. H., Moore P., Morris G. E., Alcock N. W. (1982). J. Chem. Soc., Dalton Trans..

[cit55] Bitterwolf T. E., Saygh A. A., Shade J. E., Rheingold A. L., Yap G. P. A., Lable-Sands L. M. (2000). Inorg. Chim. Acta.

[cit56] Oishi M., Oshima M., Suzuki H. (2014). Inorg. Chem..

[cit57] Oishi M., Suzuki H. (2009). Inorg. Chem..

[cit58] Oishi M., Kato T., Nakagawa M., Suzuki H. (2008). Organometallics.

[cit59] Lassalle S., Petit J., Falconer R. L., Hérault V., Jeanneau E., Thieuleux C., Camp C. (2022). Organometallics.

[cit60] Lassalle S., Jabbour R., Schiltz P., Berruyer P., Todorova T. K., Veyre L., Gajan D., Lesage A., Thieuleux C., Camp C. (2019). J. Am. Chem. Soc..

[cit61] Lassalle S., Jabbour R., Del Rosal I., Maron L., Fonda E., Veyre L., Gajan D., Lesage A., Thieuleux C., Camp C. (2020). J. Catal..

[cit62] Escomel L., Soulé N., Robin E., Del Rosal I., Maron L., Jeanneau E., Thieuleux C., Camp C. (2022). Inorg. Chem..

[cit63] Ye C. Z., Del Rosal I., Boreen M. A., Ouellette E. T., Russo D. R., Maron L., Arnold J., Camp C. (2022). Chem. Sci..

[cit64] Del Rosal I., Lassalle S., Dinoi C., Thieuleux C., Maron L., Camp C. (2021). Dalton Trans..

[cit65] Zhang Y., Roberts S. P., Bergman R. G., Ess D. H. (2015). ACS Catal..

[cit66] Hostetler M. J., Bergman R. G. (1990). J. Am. Chem. Soc..

[cit67] Hostetler M. J., Butts M. D., Bergman R. G. (1993). J. Am. Chem. Soc..

[cit68] Butts M. D., Bergman R. G. (1994). Organometallics.

[cit69] Hostetler M. J., Butts M. D., Bergman R. G. (1992). Inorg. Chim. Acta.

[cit70] Hunter N. H., Lane E. M., Gramigna K. M., Moore C. E., Thomas C. M. (2021). Organometallics.

[cit71] Maiola M. L., Buss J. A. (2023). Angew. Chem., Int. Ed..

[cit72] Deolka S., Rivada-Wheelaghan O., Aristizábal S. L., Fayzullin R. R., Pal S., Nozaki K., Khaskin E., Khusnutdinova J. R. (2020). Chem. Sci..

[cit73] Antwi-Nsiah F. H., Oke O., Cowie M. (1996). Organometallics.

[cit74] Hidalgo N., Maya C., Campos J. (2019). Chem. Commun..

[cit75] Campos J. (2017). J. Am. Chem. Soc..

[cit76] Shima T., Suzuki H. (2000). Organometallics.

[cit77] Takao T., Monoe T., Shimogawa R., Nakamura K. (2023). Organometallics.

[cit78] Bruce G. C., Knox S. A. R., Phillips A. J. (1990). J. Chem. Soc., Chem. Commun..

[cit79] O'Leary N., Miloserdov F. M., Mahon M. F., Whittlesey M. K. (2019). Dalton Trans..

[cit80] Garçon M., Mun N. W., White A. J. P., Crimmin M. R. (2021). Angew. Chem., Int. Ed..

[cit81] Alférez M. G., Moreno J. J., Hidalgo N., Campos J. (2020). Angew. Chem., Int. Ed..

[cit82] Alférez M. G., Moreno J. J., Maya C., Campos J. (2023). Dalton Trans..

[cit83] Liu S., Motta A., Mouat A. R., Delferro M., Marks T. J. (2014). J. Am. Chem. Soc..

[cit84] McInnis J. P., Delferro M., Marks T. J. (2014). Acc. Chem. Res..

[cit85] Liu S., Motta A., Delferro M., Marks T. J. (2013). J. Am. Chem. Soc..

[cit86] Wang J., Li H., Guo N., Li L., Stern C. L., Marks T. J. (2004). Organometallics.

[cit87] Ishino H., Takemoto S., Hirata K., Kanaizuka Y., Hidai M., Nabika M., Seki Y., Miyatake T., Suzuki N. (2004). Organometallics.

[cit88] Sakaba H., Ishida K., Horino H. (1998). Chem. Lett..

[cit89] Jeffery J. C., Orpen A. G., Stone F. G. A., Went M. J. (1986). J. Chem. Soc., Dalton Trans..

[cit90] Lorber C., Vendier L. (2010). Organometallics.

[cit91] Fieser M. E., Mueller T. J., Bates J. E., Ziller J. W., Furche F., Evans W. J. (2014). Organometallics.

[cit92] Evans W. J., Perotti J. M., Ziller J. W. (2005). Inorg. Chem..

[cit93] Evans W. J., Ulibarri T. A., Ziller J. W. (1991). Organometallics.

[cit94] Tsai C. C., Shih W. C., Fang C. H., Li C. Y., Ong T. G., Yap G. P. A. (2010). J. Am. Chem. Soc..

[cit95] Shih W. C., Chen W. C., Lai Y. C., Yu M. S., Ho J. J., Yap G. P. A., Ong T. G. (2012). Org. Lett..

[cit96] Zhang T., Luan Y. X., Lam N. Y. S., Li J. F., Li Y., Ye M., Yu J. Q. (2021). Nat. Chem..

[cit97] Okumura S., Tang S., Saito T., Semba K., Sakaki S., Nakao Y. (2016). J. Am. Chem. Soc..

[cit98] Yang L., Semba K., Nakao Y. (2017). Angew. Chem., Int. Ed..

[cit99] Okumura S., Nakao Y. (2017). Org. Lett..

[cit100] Nakao Y., Yamada Y., Kashihara N., Hiyama T. (2010). J. Am. Chem. Soc..

[cit101] Okumura S., Komine T., Shigeki E., Semba K., Nakao Y. (2018). Angew. Chem., Int. Ed..

[cit102] Hicks J., Mansikkamäki A., Vasko P., Goicoechea J. M., Aldridge S. (2019). Nat. Chem..

[cit103] Luan Y. X., Ye M. (2022). Chem. Commun..

[cit104] Graziano B. J., Vollmer M. V., Lu C. C. (2021). Angew. Chem., Int. Ed..

[cit105] Gorgas N., White A. J. P., Crimmin M. R. (2022). J. Am. Chem. Soc..

[cit106] Gorgas N., White A. J. P., Crimmin M. R. (2022). Chem. Commun..

[cit107] Liu J., Li Y., Jiang J., Liu Y., Ke Z. (2021). ACS Catal..

[cit108] Cao Y., Shih W. C., Bhuvanesh N., Zhou J., Ozerov O. V. (2021). Chem. Sci..

[cit109] Shih W. C., Ozerov O. V. (2017). J. Am. Chem. Soc..

[cit110] Stadler B., Gorgas N., White A. J. P., Crimmin M. R. (2023). Angew. Chem..

[cit111] Gorgas N. , Stadler B., White A. J. P. and Crimmin M. R., *ChemRxiv*, 2023

[cit112] Hara N., Uemura N., Nakao Y. (2021). Chem. Commun..

[cit113] Hara N., Aso K., Li Q. Z., Sakaki S., Nakao Y. (2021). Tetrahedron.

[cit114] Seki R., Hara N., Saito T., Nakao Y. (2021). J. Am. Chem. Soc..

[cit115] Li Q. Z., Hara N., Semba K., Nakao Y., Sakaki S. (2022). Top. Catal..

[cit116] Yamada R., Iwasawa N., Takaya J. (2019). Angew. Chem., Int. Ed..

[cit117] Liu H. Y., Neale S. E., Hill M. S., Mahon M. F., McMullin C. L. (2023). Chem. Sci..

[cit118] Gentner T. X., Evans M. J., Kennedy A. R., Neale S. E., McMullin C. L., Coles M. P., Mulvey R. E. (2022). Chem. Commun..

[cit119] Hicks J., Vasko P., Heilmann A., Goicoechea J. M., Aldridge S. (2020). Angew. Chem., Int. Ed..

[cit120] Hicks J., Vasko P., Goicoechea J. M., Aldridge S. (2018). Nature.

[cit121] Hicks J., Vasko P., Goicoechea J. M., Aldridge S. (2019). J. Am. Chem. Soc..

[cit122] Muhr M., Bühler R., Liang H., Gilch J., Jandl C., Kahlal S., Saillard J. Y., Gemel C., Fischer R. A. (2022). Chem. – Eur. J..

[cit123] Bajo S., Alcaide M. M., López-Serrano J., Campos J. (2021). Chem. – Eur. J..

[cit124] Hadlington T. J., Kostenko A., Driess M. (2020). Chem. – Eur. J..

[cit125] Mai J., Rösch B., Patel N., Langer J., Harder S. (2023). Chem. Sci..

[cit126] Mulvey R. E. (2009). Acc. Chem. Res..

[cit127] Robertson S. D., Uzelac M., Mulvey R. E. (2019). Chem. Rev..

[cit128] Hevia E., Gallagher D. J., Kennedy A. R., Mulvey R. E., O'Hara C. T., Talmard C. (2004). Chem. Commun..

[cit129] Andrikopoulos P. C., Armstrong D. R., Barley H. R. L., Clegg W., Dale S. H., Hevia E., Honeyman G. W., Kennedy A. R., Mulvey R. E. (2005). J. Am. Chem. Soc..

[cit130] Carrella L. M., Clegg W., Graham D. V., Hogg L. M., Kennedy A. R., Klett J., Mulvey R. E., Rentschler E., Russo L. (2007). Angew. Chem., Int. Ed..

[cit131] Ballmann G. M., Gentner T. X., Kennedy A. R., Hevia E., Mulvey R. E. (2022). Chem. – Eur. J..

[cit132] Nobuto D., Uchiyama M. (2008). J. Org. Chem..

[cit133] Alborés P., Carrella L. M., Clegg W., García-Álvarez P., Kennedy A. R., Klett J., Mulvey R. E., Rentschler E., Russo L. (2009). Angew. Chem., Int. Ed..

[cit134] Logallo A., Mu M., García-Melchor M., Hevia E. (2022). Angew. Chem., Int. Ed..

[cit135] Logallo A., Hevia E. (2023). Chem. Commun..

[cit136] Gil-Negrete J. M., Hevia E. (2021). Chem. Sci..

[cit137] Logallo A., Hevia E. (2022). Chimia.

[cit138] Judge N., Logallo A., Hevia E. (2023). Chem. Sci..

[cit139] Maddock L. C. H., Nixon T., Kennedy A. R., Probert M. R., Clegg W., Hevia E. (2018). Angew. Chem., Int. Ed..

[cit140] Judge N. R., Hevia E. (2023). Angew. Chem., Int. Ed..

[cit141] Maddock L. C. H., Mu M., Kennedy A. R., García-Melchor M., Hevia E. (2021). Angew. Chem., Int. Ed..

[cit142] Liu L., Corma A. (2023). Chem. Rev..

[cit143] Escomel L., Abbott D., Mougel V., Veyre L., Thieuleux C., Camp C. (2022). Chem. Commun..

